# A Local Counter-Regulatory Motif Modulates the Global Phase of Hormonal Oscillations

**DOI:** 10.1038/s41598-017-01806-0

**Published:** 2017-05-09

**Authors:** Dong-Ho Park, Taegeun Song, Danh-Tai Hoang, Jin Xu, Junghyo Jo

**Affiliations:** 1 Asia Pacific Center for Theoretical Physics, Pohang, Gyeongbuk 37673 Korea; 2 Laboratory of Biological Modeling, National Institute of Diabetes and Digestive and Kidney Diseases, National Institutes of Health, Bethesda, Maryland 20892 United States of America; 3 Department of Natural Sciences, Quang Binh University, Dong Hoi, Quang Binh 510000 Vietnam; 4 Department of Physics, Pohang University of Science and Technology, Pohang, Gyeongbuk 37673 Korea

## Abstract

Counter-regulatory elements maintain dynamic equilibrium ubiquitously in living systems. The most prominent example, which is critical to mammalian survival, is that of pancreatic α and β cells producing glucagon and insulin for glucose homeostasis. These cells are not found in a single gland but are dispersed in multiple micro-organs known as the islets of Langerhans. Within an islet, these two reciprocal cell types interact with each other and with an additional cell type: the δ cell. By testing all possible motifs governing the interactions of these three cell types, we found that a unique set of positive/negative intra-islet interactions between different islet cell types functions not only to reduce the superficially wasteful zero-sum action of glucagon and insulin but also to enhance/suppress the synchronization of hormone secretions between islets under high/normal glucose conditions. This anti-symmetric interaction motif confers effective controllability for network (de)synchronization.

## Introduction

Living systems must maintain internal homeostasis in the face of external perturbations^1^. The endocrine system orchestrates dynamic equilibrium via long-range messengers known as hormones. Most physiological processes are controlled by negative feedback and antagonistic pairs of hormones, such as insulin/glucagon for glucose homeostasis^2^, calcitonin/parathyrin for calcium homeostasis^3^, and leptin/ghrelin for energy homeostasis^4^. Hormones also exhibit temporal oscillations^5, 6^. The mechanistic reason for wave-like hormonal information broadcasting is less well known, although both a wave’s amplitude and phase encode information. In addition to amplitude modulation, the relative phase coordination between different hormones and phase synchronization between different sources (cells or tissues) of the same hormones could have functional implications. Here, we focus on the example of phase modulation in glucose homeostasis, a much-studied case due to the critical importance of glucose homeostasis in human survival.

**Figure 1:**
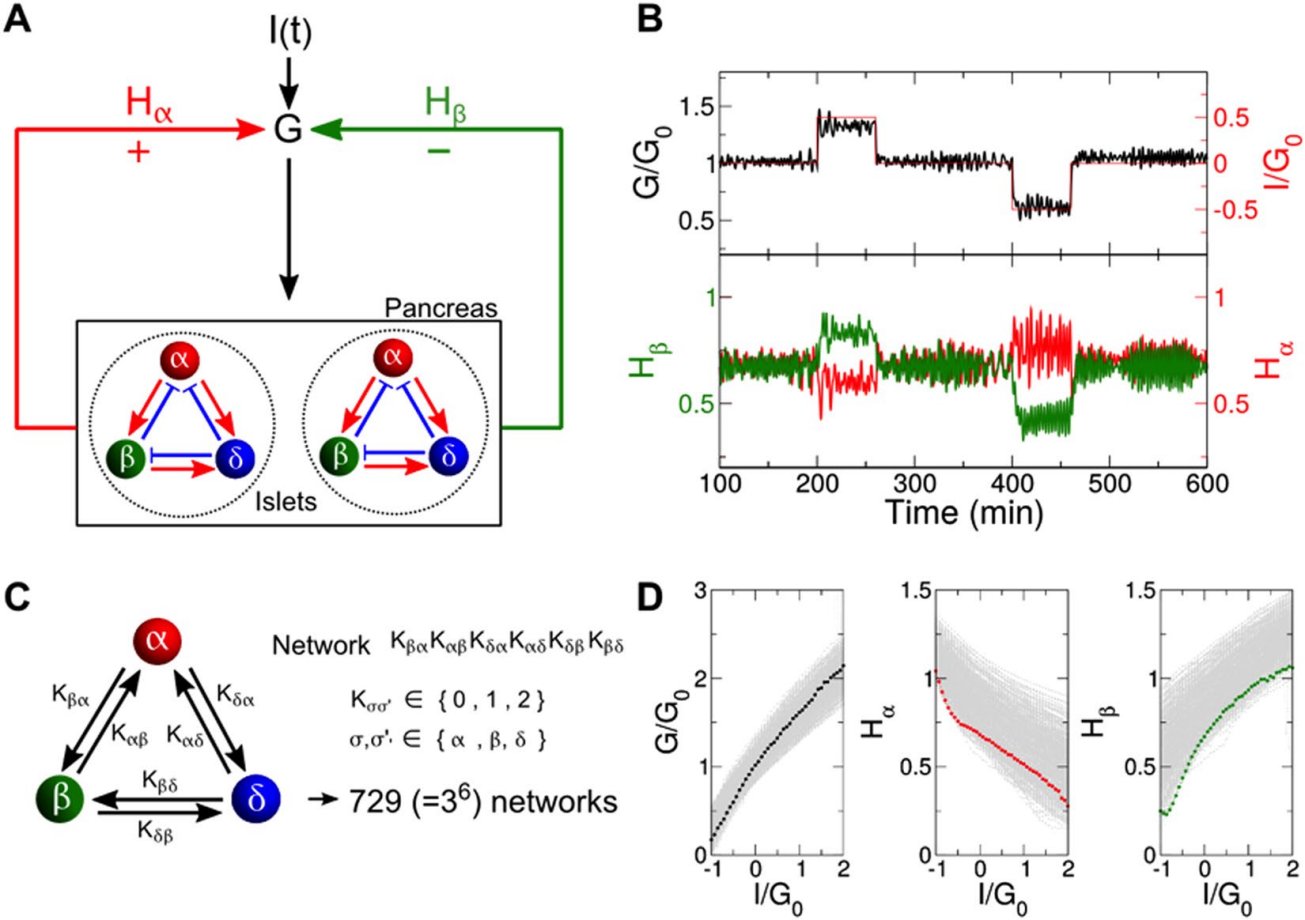
Glucose regulation and islet cell network. (**A**) Schematic diagram of glucose regulation by pancreatic islets. Endocrine α, β, and δ cells in the pancreatic islets monitor and regulate blood glucose concentration, *G*, perturbed by external input, *I*. Glucagon, *H*_*α*_, secreted by α cells increases *G*, while insulin, *H*_*β*_, secreted by β cells decreases *G*. (**B**) Given the external glucose input, *I*(*t*) (red line in upper plot), glucose *G* (solid black line) is regulated by two counter-regulatory hormones: *H*_*α*_ (red line in lower plot) and *H*_*β*_ (green line). Note that *G*_0_ is the normal glucose concentration in the absence of an input (*I* = 0). Network 121212 is used. (**C**) Network annotation for describing the interactions between islet cells. The interaction signs of *A*_*βα*_, *A*_*αβ*_, *A*_*δα*_, *A*_*αδ*_, *A*_*δβ*_, and *A*_*βδ*_ can take zero (0), positive (1), or negative (2) values. Following this notation, network 121212 represents the native network of islet cells. (**D**) Stationary glucose concentration and the corresponding hormone consumption for various external glucose inputs. Negative/positive inputs, *I*, induce low/high glucose conditions. A total of 729 networks were considered: network 121212 (black, red, green) and the other networks (gray). See Section 2 in the Supplementary Material for the details of the model and standard parameter values.

Glucose, which is a primary energy source in the body, is mainly regulated by glucagon and insulin secreted by α and β cells in the pancreas. Glucagon increases blood glucose by stimulating the breakdown of glycogen into glucose in the liver, while insulin decreases blood glucose by stimulating the synthesis of glycogen from glucose in the liver and clearing glucose into peripheral tissues. Pancreatic α and β cells are clustered together with an additional cell type, the δ cell, to form micro-organs known as the islets of Langerhans. Approximately one million islets are scattered throughout the human pancreas. Pancreatic α, β, and δ cells generate pulses of glucagon, insulin, and somatostatin, respectively. For the effective regulation of glucose homeostasis, coordination between insulin and glucagon pulses and between different islets is required. Approximate out-of-phase coordination of insulin and glucagon pulses has been observed in *in vitro* ^7^ and *in vivo* experiments^8^. This coordination implies interactions between islet cells. Indeed, it has long been observed that paracrine factors from α cells provide positive feedback to hormone secretion by β and δ cells, while δ cells provide negative feedback to α and β cells^9^. Furthermore, β cells exert negative feedback on α cells, but positive feedback on δ cells, which was recently confirmed^10^ (See Fig. 1A). The rationale for this set of interactions is a long-standing puzzle^11^. A separate and equally important form of coordination is inter-islet synchronization. The independence or coherence of hormone secretions from one million islets must have a large impact on human physiology. It has been implicitly assumed that islets are always synchronized to produce oscillatory hormone profiles in the blood, as the alternative would be a flat hormone profile due to the asynchronous action of one million islets. However, this assumption must be reconsidered because recent results raise the possibility that islets are not functionally identical^12^.

Here, we model the intra- and inter-islet network, and examine the glucose regulation by the hierarchical islet network. The native intra-islet network is composed of anti-symmetric interactions between α, β, and δ cells. We consider all possible alternatives to the native network motif, and examine their phase coordination between insulin and glucagon pules and between islets and their glucose regulation. Then, we demonstrate that the native motif is one of the most effective motifs that stably regulate glucose levels with minimal hormone consumption, and flexibly control inter-islet synchronization depending on glucose levels.

## Intra-islet network and glucose homeostasis

To probe how unique the native intra-islet network is for controlling inter-islet synchronization and stably regulating glucose levels, we formulate the glucose-regulation system on the basis of four key observations:

(i) Islet cells produce hormone pulses depending on glucose levels^13^;
(ii) Islet cells interact with each other with a special symmetry^9^;
(iii) Insulin and glucagon regulate glucose levels^14^;
(iv) Oscillatory glucose levels entrain islets to produce synchronous hormone pulses^15–17^.

The spontaneous hormone pulses and their interactions (i, ii) can be simply described by a coupled oscillator model^18^. Here, we extend the model for multiple islets and explicitly consider the glucose regulation and entrainment (iii, iv). By considering glucose as a dynamic variable in the whole system, glucose stimulates islets, while islets regulate glucose.

The spontaneous hormone pulses or oscillations of α, β, and δ cells can be described by the amplitude and phase of a generic oscillator^19^:

1 $${\dot{r}}_{n\sigma }={\tau }_{r}^{-1}[{f}_{\sigma }(G)-{r}_{n\sigma }^{2}]{r}_{n\sigma },$$

2 $${\dot{\theta }}_{n\sigma }={\omega }_{n\sigma }-{g}_{\sigma }(G)\cos \,{\theta }_{n\sigma }$$

for the *σ* ∈ {*α*, *β*, *δ*} cell type in the *n*th islet. The differential equations generate oscillations with a stationary amplitude of ${r}_{n\sigma }=\sqrt{{f}_{\sigma }}$ and an intrinsic phase velocity of *ω*_*nσ*_. We focus on *slow* hormone oscillation with a period of 2*π*/*ω*_*nσ*_ = 5 ± 1 minutes^2^. A characteristic time constant, *τ*_*r*_ = 1 min, is introduced to match the time scale for amplitude and phase dynamics. Given the amplitude and phase, the hormone secretion from the *σ* cell in the *n*th islet is defined as ${H}_{n\sigma }\equiv {r}_{n\sigma }(\cos \,{\theta }_{n\sigma }+1)$, where the phase *θ*_*nσ*_ = 0 and *π* represent maximal and basal secretion, respectively. The cosine has been shifted by unity to prevent negativity of hormone secretion. The amplitude modulation, *f*_*σ*_(*G*), depends on the glucose concentration, *G*. Briefly *f*_*α*_ and *f*_*β*_ are decreasing and increasing functions of *G*, respectively, as α cells secrete glucagon at low glucose levels, while β cells secrete insulin at high glucose levels^20^. Furthermore, the phase modulation, *g*_*σ*_(*G*), accounts for the fact that hormone pulses are not pure sine waves. They modulate the duration of active and silent phases depending on the glucose concentration. Islet Ca^2+^ oscillations imply that insulin pulses have longer active phases at higher glucose levels^21–23^ (See Section 1 in Supplementary Information for the detailed description for the model including *f*_*σ*_(*G*) and *g*_*σ*_(*G*)).

Each islet responds to a global glucose concentration, *G*, and secretes hormones accordingly (Fig. 1A). The total glucagon, $N\cdot {H}_{\alpha }={\sum }_{n=1}^{N}{H}_{n\alpha }$, from *N* islets increases *G*, while the total insulin, $N\cdot {H}_{\beta }={\sum }_{n=1}^{N}{H}_{n\beta }$, decreases *G*. Unlike the positive glucose flux associated with glucagon, the negative glucose flux associated with insulin is proportional to the present glucose concentration. The following equation summarizes glucose regulation:

3 $$\dot{G}=\lambda N({G}_{0}{H}_{\alpha }-G\,{H}_{\beta })+I(t),$$

where *λ* represents the effectiveness of hormone action for glucose regulation, and *I*(*t*) represents external glucose inputs reflecting food intake (positive input) or exogenous insulin injection (negative input). Here, we introduce a constant parameter (*G*_0_ = 7 mM) to match the scale for glucagon and insulin action. In the absence of glucose inputs (*I* = 0), a normal glucose concentration is approximated by *G* ≈ *G*_0_ because the glucagon and insulin amplitudes balance (*r*_*nα*_ ≈ *r*_*nβ*_) at *G*_0_ and vanish the glucose regulation ($\dot{G}=0$). Given positive/negative glucose inputs, the islet system produces more insulin/glucagon to less perturb the normal glucose concentration (Fig. 1B). Here, islets do not interact directly with each other, but the total hormonal secretions modulate glucose, which in turn affects each islet. For example, β cells secrete insulin, which reduces glucose and thereby inhibits β cells and stimulates α cells. This is a kind of mean field model in which individual islets interact indirectly though the mean field of global glucose. Consequently, spontaneous hormone oscillations lead to oscillations of glucose. The amplitude variation in the glucose concentration can contribute to entraining islets to exhibit similar phases^24, 25^. *Glucose entrainment* is one mechanistic explanation for inter-islet synchronization with the mechanism mediated by the neural pacemaker within the pancreas^13^.

Now, we consider the intra-islet interaction between α, β, and δ cells and their roles for regulating glucose homeostasis. First, using a complex variable, ${Z}_{n\sigma }\equiv {r}_{n\sigma }{e}^{i{\theta }_{n\sigma }}$, and its complex conjugate, ${Z}_{n\sigma }^{\ast }$, we combine the amplitude and phase dynamics in Eqs (1) and (2): ${\dot{Z}}_{n\sigma }={J}_{n\sigma }\,{Z}_{n\sigma }$, where ${J}_{n\sigma }\equiv {f}_{\sigma }-{Z}_{n\sigma }{Z}_{n\sigma }^{\ast }+i{\omega }_{n\sigma }-i{g}_{\sigma }({Z}_{n\sigma }+{Z}_{n\sigma }^{\ast }){(4{Z}_{n\sigma }{Z}_{n\sigma }^{\ast })}^{-1/2}$. Then, in the presence of the intra-islet interaction, the islet model is written as follows:

4 $${\dot{Z}}_{n\sigma }={J}_{n\sigma }\,\,{Z}_{n\sigma }+K\sum _{\sigma \text{'}}{A}_{\sigma \sigma \text{'}}\,{Z}_{n\sigma \text{'}},$$

where *K* represents the interaction strength, and the adjacency matrix, *A*_*σσ*′_, represents the interaction signs from *σ*′ cell to *σ* cell within each islet. Positive/negative interaction leads the amplitude and phase of *σ* cell to be positively/negatively correlated to the amplitude and phase of *σ*′ cell. For example, positive interaction from *σ*′ cell stimulates neighboring *σ* cell to produce larger oscillation and pull its phase to be in phase with *σ*′ cell. Ignoring self-interactions (*A*_*σσ*_ = 0), a total of 729 (=3^6^) networks are possible with either positive, no, or negative interactions for each link (Fig. 1C). We then conducted glucose regulation simulations with various glucose stimuli, and obtained the corresponding glucose level and glucagon and insulin secretions for all possible networks (Fig. 1D). All of the networks could reasonably control the glucose stimuli by balancing the antagonistic hormones glucagon and insulin. Interestingly, native islet network 121212 emerged as one of the most efficient networks requiring minimal hormone secretion to achieve glucose regulation (See Section 2 in the Supplementary Information for the parameter independence of our conclusion).

## Effective networks for minimal hormone consumption

**Figure 2:**
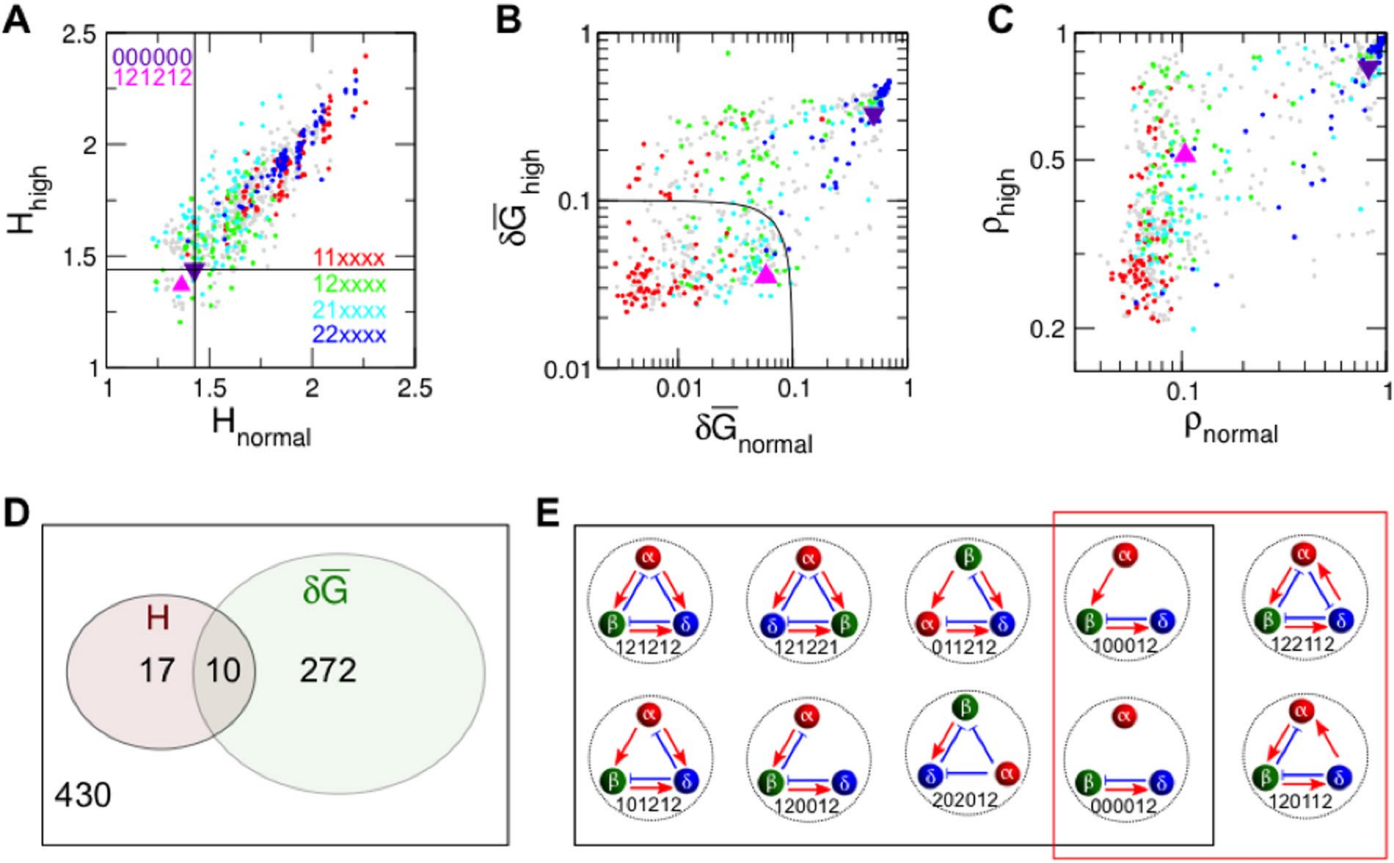
Efficient hormone consumption and glucose regulation. (**A**) Hormone consumption, $H\equiv {H}_{\alpha }+{H}_{\beta }$, of 729 networks under normal (*I* = 0) and high (*I* = 2*G*_0_) glucose conditions: networks 121212 (pink triangle, native interaction between islet cells), 000000 (purple inverted triangle, no interaction), 11xxxx (red circles, mutual activation between α and β cells), 12xxxx (green circles, native asymmetric interaction), 21xxxx (cyan circles, inverse asymmetric interaction), 22xxxx (blue circles, mutual inhibition), and the others (gray circles). Network 000000 is located at the crossing of vertical and horizontal black lines. (**B**) Temporal fluctuations of glucose, normalized according to the absolute glucose concentration, $\delta \bar{G}\equiv \delta G/G$, under normal/high glucose conditions. Black lines separate the networks that produce small glucose fluctuations ($\delta {\bar{G}}_{{\rm{normal}}}^{2}+\delta {\bar{G}}_{{\rm{high}}}^{2} < {0.1}^{2}$). (**C**) Degree of synchronization between islets under normal/high glucose conditions. The time-averaged synchronization index, *ρ* = 1/0, represents perfect synchronization/independence between islets. (**D**) Ten effective networks satisfy the two criteria of minimal hormone consumption and small glucose fluctuations inside the black lines in (**A**) and (**B**). (**E**) Topologies of the ten effective networks with their annotations. Positive/negative interactions are represented by red/blue bar-headed arrows, respectively. A black rectangle groups subsets and transformations of network 121212, while a red rectangle groups subsets of network 122112. To highlight this, networks are plotted with fixed arrow positions, not cell positions.

In glucose regulation balanced by glucagon and insulin, some wasteful cosecretion of the antagonistic hormones is possible. Thus, we examined the hormone consumption, *H*_*α*_ + *H*_*β*_, of the 729 networks under normal (*I* = 0) and high (*I* = 2*G*_0_) glucose conditions for 300 min and 200 min, respectively (See Section 3 in the Supplementary Information for the total hormone consumption including somatostatin and temporal behaviors of three hormones). After removing initial equilibration periods, we calculated average hormone consumptions for the last *T* = 100 min: ${H}_{{\rm{normal}}}={T}^{-1}{\int }_{200}^{200+T}[{H}_{\alpha }(t)+{H}_{\beta }(t)]dt$ and ${H}_{{\rm{high}}}={T}^{-1}{\int }_{400}^{400+T}[{H}_{\alpha }(t)+{H}_{\beta }(t)]dt$ (Fig. 2A). As α and β cells are key components of glucose regulation, first we focused on the mutual interaction between these cells by comparing four groups of networks: (i) mutual activation networks (11xxxx); (ii) mutual inhibition networks (22xxxx); (iii) native asymmetric networks (12xxxx); and (iv) inverse asymmetric networks (21xxxx). We expected that the mutual inhibition networks could effectively prevent the cosecretion of insulin and glucagon. However, the mutual activation/inhibition networks generally consume larger amounts of hormones because the mutual interaction has the same sign and construct positive feedback loops (α → β → α and β → α → β) regardless of the signs (positive/negative). The mutual activation/inhibition networks have positive coupling terms, ${A}_{\alpha \beta }\,\cos ({\theta }_{n\beta }-{\theta }_{n\alpha })$ and ${A}_{\beta \alpha }\,\cos ({\theta }_{n\alpha }-{\theta }_{n\beta })$, in the amplitude component of Eq. (4), which increase both amplitudes, *r*_*nα*_ and *r*_*nβ*_. In contrast, the asymmetric networks consume smaller amounts of hormones than the symmetric networks. In particular, 12xxxx networks consume lower levels of hormones than 21xxxx networks at high glucose because α/β cells are successfully suppressed/enhanced to decrease glucose levels.

Next, we checked the robustness of glucose regulation by quantifying the fluctuations of the glucose concentration: $\delta {G}^{2}={T}^{-1}{\int }_{{t}_{0}}^{{t}_{0}+T}{[G(t)-\overline{G}]}^{2}dt$, where $\overline{G}={T}^{-1}{\int }_{{t}_{0}}^{{t}_{0}+T}G(t)dt$. The corresponding fluctuations under normal and high glucose conditions were calculated with *t*_0_ = 200 min and *t*_0_ = 400 min, respectively (Fig. 2B). The amplitude variations in the glucose concentration can entrain islets to produce synchronized hormone pulses. Then, the large variations in synchronized hormones from the entrained islets may cause larger-amplitude variations in the glucose concentration. Thus, a strong correlation is expected between glucose fluctuations (Fig. 2B) and inter-islet synchronization (Fig. 2C). Here, to quantify the phase synchronization between islets, we adopted the usual synchronization index for oscillators, ${\rho }_{\sigma }{e}^{i{\Theta }_{\sigma }}={N}^{-1}{\sum }_{n=1}^{N}{e}^{i{\theta }_{n\sigma }}$, where *ρ*_*σ*_ measures the degree of synchronization between *σ* cells located in different islets (0/1 for perfect desynchronization/synchronization), and Θ_*σ*_ captures their average phase at a certain time. In particular, we used their time-averaged index, $\rho ={(3T)}^{-1}{\int }_{{t}_{0}}^{{t}_{0}+T}[{\rho }_{\alpha }(t)+{\rho }_{\alpha }(t)+{\rho }_{\alpha }(t)]\,\,dt$ with *t*_0_ = 200 min for *ρ*_normal_ and *t*_0_ = 400 min for *ρ*_high_. In general, 11xxxx networks show small glucose fluctuations and low inter-islet synchronization, while 22xxxx networks show large glucose fluctuations and high inter-islet synchronization. Mutual activation networks exhibit *frustration* for the coordination between α and β cells: (internal) mutual positive interactions lead them to display in-phase coordination, while (external) glucose leads them to present out-of-phase coordination. The frustration hinders different islets from generating coherent behavior under glucose entrainment. On the contrary, mutual inhibition networks do not experience frustration, and every islet is easily entrained to external glucose with out-of-phase coordination between α and β cells.

This strong correlation between glucose fluctuations and inter-islet synchronization is not always found in networks 12xxxx and 21xxxx. At normal glucose concentrations, these networks show small glucose fluctuations and low inter-islet synchronization. However, at high glucose levels, the asymmetric networks show a wide range of glucose fluctuations and inter-islet synchronization. Some networks, including the native islet network 121212, exhibit small glucose fluctuations with high inter-islet synchronization, which seems counterintuitive because the large variations in coherent hormone pulses from synchronized islets can lead to large fluctuations of glucose concentrations governed by Eq. (3). Furthermore, the out-of-phase coordination between *H*_*α*_ and *H*_*β*_ at high glucose levels can amplify glucose fluctuations by alternatively increasing and decreasing glucose concentrations. The network 121212 generates synchronous hormone pulses of *H*_*nα*_ and *H*_*nβ*_, and has the out-of-phase coordination between their average glucagon *H*_*α*_ and insulin *H*_*β*_. Nevertheless, it does not induce large glucose fluctuations because the combined phase of the glucose and insulin action (*G* · *H*_*β*_) is not out of phase with *H*_*α*_. The combined phase, not the pure insulin phase, regulates $\dot{G}$ in Eq. (3). The effective phase coordination between glucose and insulin allows $\dot{G}$ to remain negligibly small in stationary states. Thus, network 121212 can achieve coherent hormone pulses of synchronized islets but maintain small glucose fluctuations at high glucose levels.

Effective homeostatic networks should tightly regulate the glucose concentration with small fluctuations by consuming minimal amounts of hormones. Among a total of 729 possible networks, we sorted out the effective networks that consume smaller amounts of hormones than network 000000 (no interaction between islet cells) and show small glucose fluctuations (*δG*/*G* < 0.1) (Fig. 2D). The applied criteria identified ten effective networks (Fig. 2E and Table 1). Indeed, these networks are subsets or transformations of network 121212 or 122112. In other words, if one removes some links or changes cell names from 121212 or 122112, the remaining eight networks can be obtained. We noted that the asymmetric interactions between islet cells are prevalent in the ten effective networks, and mutual activation/inhibition never occurs between any pair of cells. In particular, the asymmetric interaction between β and δ cells are well conserved: nine effective networks have the xxxx12 type in which β cells activate δ cells while δ cells suppress β cells. The ten effective networks could stably regulate glucose with minimal consumption of two antagonistic hormones, insulin and glucagon. Their somatostatin consumption was also relatively minimal (Table S2). Moreover, they showed controllable inter-islet synchronization with low/high synchronization index at normal/high glucose (Table 1). Among them, the fully connected networks 121212, 121221, and 122112 showed higher inter-islet synchronization at high glucose. Here, network 121221 is topologically equivalent to network 121212. The name switch between β and δ cells can transform network 121221 to network 121212. On the other hand, networks 121212 and 122112 are topologically distinct. Network 121212 exhibits distinguishable cells: one cell suppresses the other two; one cell enhances the other two; and one cell suppresses one and enhances one. However, network 122112 shows indistinguishable cells: every cell suppresses and enhances the others. In other words, if one hides cell names, one cannot distinguish between cells. Here, network 122112 may require more complex sets of receptors to realize the interaction topology because one cell must affect the other two cells differently. Finally, we have also examined the phase coordination between three hormones for the ten effective networks. Networks 121212 and 120012 clearly reproduced the out-of-phase coordination between insulin and glucagon pulses, and the in-phase coordination between insulin and somatostatin (Fig. S9), as experimentally observed by Hellman *et al*.^7^ In summary, network 121212 outstands in many aspects such as minimal hormone consumption, stable glucose regulation, controllable inter-islet synchronization, and clear phase coordination between three hormones.

**Table 1:** Hormone consumption, glucose fluctuation, and inter-islet synchronization of ten effective networks under normal and high glucose conditions.

#	Network	*H*_normal_	*H*_high_	$\delta {\bar{G}}_{{\rm{normal}}}$	$\delta {\bar{G}}_{{\rm{high}}}$	*ρ*_normal_	*ρ*_high_
1	000012	1.357	1.288	0.048	0.052	0.089	0.319
2	011212	1.292	1.351	0.012	0.048	0.065	0.335
3	202012	1.329	1.299	0.039	0.040	0.116	0.309
4	100012	1.368	1.261	0.011	0.039	0.063	0.321
5	101212	1.295	1.395	0.015	0.034	0.082	0.343
6	120012	1.244	1.383	0.041	0.041	0.090	0.373
7	120112	1.418	1.311	0.011	0.032	0.073	0.419
8	122112	1.359	1.204	0.076	0.031	0.144	0.466
9	121221	1.360	1.378	0.085	0.038	0.171	0.475
10	121212	1.365	1.369	0.058	0.035	0.104	0.513

## Controlling inter-islet synchronization

**Figure 3:**
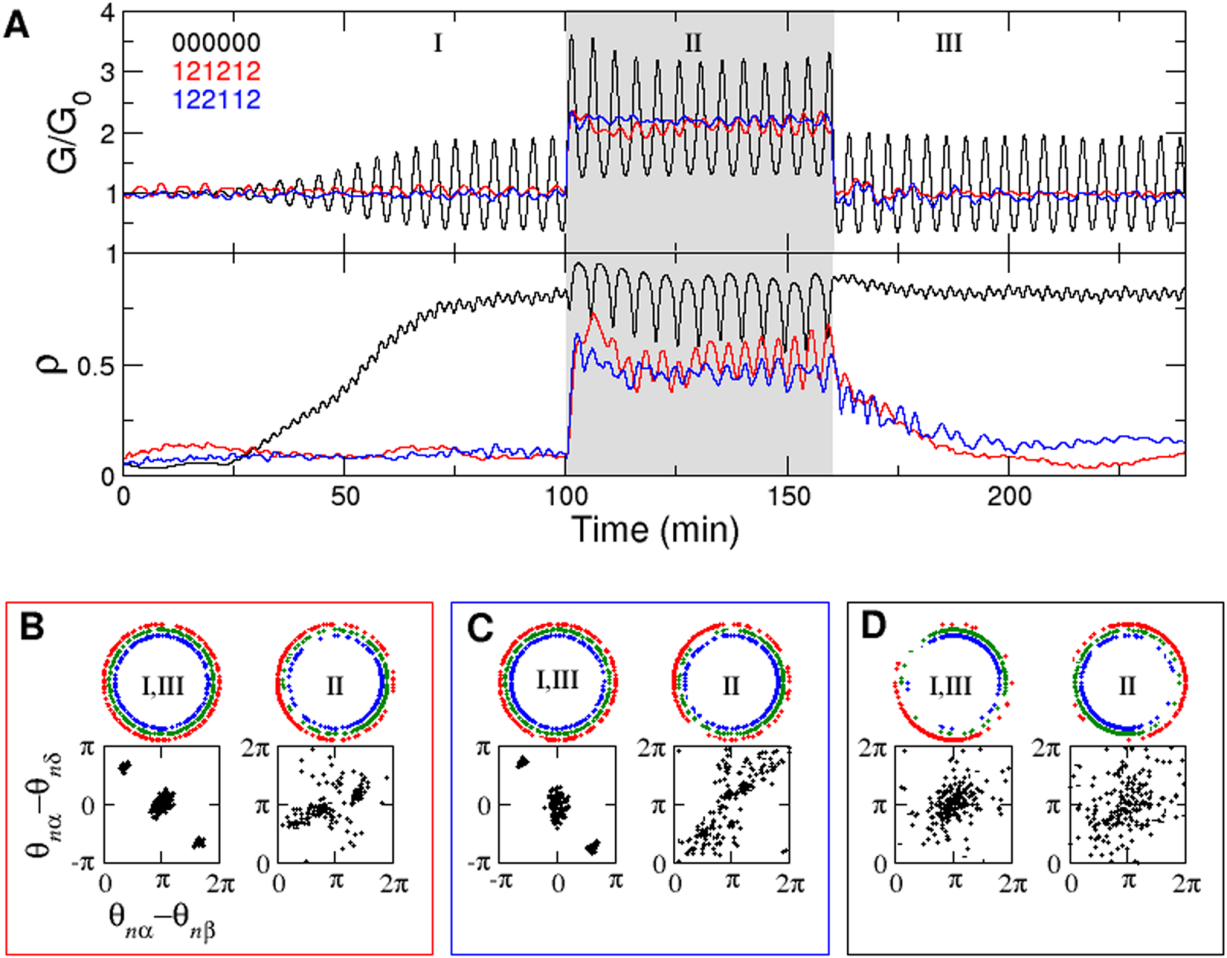
Controllable inter-islet synchronization and phase coordination between islet cells. (**A**) Glucose regulation and inter-islet synchronization for networks 121212 (red), 122112 (blue) and 000000 (black), given the external glucose input (*I* = 2*G*_0_) during 100 < *t* < 160. Phase snapshots of α, β, and δ cells under different glucose conditions (regimes I, II, and III) for (**B**) networks 121212, (**C**) 122112, and (**D**) 000000. Upper panel: absolute phases (*θ*_*nα*_, *θ*_*nβ*_, *θ*_*nδ*_) of α (red), β (green), and δ (blue) cells in the pancreas consisting of 200 islets. Lower panel: phase differences (*θ*_*nα*_−*θ*_*nβ*_, *θ*_*nα*_−*θ*_*nδ*_). The axis range was adjusted to show distinct attractors considering 2π periodicity.

The effective networks display the special feature of controllable inter-islet synchronization depending on glucose conditions. In contrast, although network 000000 (no interaction between islet cells) consumes relatively small amount of hormones among 729 networks, it exhibits a serious problem in that islets are easily entrained to glucose oscillation and synchronized with each other (Fig. 3A). Then, the synchronized hormone secretion amplifies glucose fluctuations. In contrast, networks 121212 and 122112 tightly regulate minimal glucose fluctuations, especially under normal glucose concentrations. Hence, we asked how the effective networks control inter-islet synchronization.

We found that network 121212 and 122112 display different numbers of attractors in the phase dynamics in Eq. (4) under different glucose conditions. They exhibit triple attractors of phase differences (${\theta }_{n\alpha }-{\theta }_{n\beta },\,{\theta }_{n\alpha }-{\theta }_{n\delta }$) at normal glucose levels, while the distinction between the attractors vanishes at high glucose levels (Fig. 3B and C; See Section 4 in the Supplementary Information for the analysis). Thus, different islets sit on different attractors at normal glucose levels. This causes different islets to present different phase coordination between islet cells. This heterogeneous phase coordination hinders islets from being synchronized. With high glucose levels, however, the triple attractors vanish, and islets do not present clearly distinct phase coordination between islet cells. Then, islets become easily synchronized without resistance to glucose entrainment. This scenario is exactly the same under low glucose conditions (See Section 5 in Supplementary Information). Unlike these effective networks, network 000000 shows no distinct attractors, regardless of glucose conditions (Fig. 3D), and glucose oscillations can easily entrain islets to be synchronized. Therefore, we conclude that inter-islet desynchronization at normal glucose levels is an active process that is resistant to glucose entrainment by generating multiple attractors through its dynamics. We also confirmed the controllability of inter-islet synchronization in the population model in which each islet is composed of populations of islet cells (See Section 6 in Supplementary Information).

## Discussion

Pancreatic islets secrete pulsatile hormones to regulate glucose homeostasis. Each islet is composed of α, β, and δ cells that interact with each other with a unique symmetry. We asked how special the islet-cell interaction is for coordinating hormone secretions from multiple islets and effectively regulating glucose homeostasis. Here, we have revealed the link between the intra-islet network and inter-islet synchronization, both of which have been long-standing puzzles in islet biology. First, the anti-symmetric interactions between α, β, and δ cells are unique in being able to prevent the wasteful zero-sum counter-regulatory actions of glucagon and insulin for glucose regulation. More importantly, the intra-islet network contributes to controlling the synchronization between islets in the pancreas, and the phase coordination between hormones. The multiplicity of islets in the pancreas allows signal amplification and suppression, once the coherence/independence of one million islets is controllable. The anti-symmetric regulatory motif embodies the potential of phase modulation in biological oscillations.

Rhythms and their synchronization have been an important issue in living and man-made systems^26–28^. Synchronization is not always desirable. For example, hypersynchronous neuronal activities can cause epileptic seizures in the brain^29^. Therefore, desynchronization and its controllability have been highlighted recently^30–33^. Here, pancreatic islets showed an interesting strategy for controlling synchronization. They exist as multiple micro-organs instead of a single gigantic organ such as liver, lung, and heart. This structural design may be advantageous for generating asynchronous hormone secretion. However, once required, islets can also generate synchronous hormone secretion through the glucose entrainment mechanism. Finally, since the Kuramoto model has been extensively studied for understanding synchronization phenomena^19, 26^, our study provided an explicit biological origin of the phenomenological model.

We formulated a minimal model for describing the loop of hormone secretion and glucose regulation. The model incorporated four basic observations: (i) glucose-dependent pulsatile hormone secretion; (ii) paracrine interactions between islet cells; (iii) glucose regulation by insulin and glucagon; and (iv) glucose entrainment of multiple islets. This model can provide a platform for simulating glucose regulation in various conditions. We have simulated the glucose regulation under noisy or oscillatory glucose infusions (See Sections 7 and 8 in Supplementary Information). The noisy glucose infusion lead glucose levels more fluctuating, and it did not change our conclusion about the effectiveness of the network 121212. Next, the oscillatory glucose infusion could entrain hormone secretion to follow the glucose rhythm, if the oscillation amplitude of external glucose is sufficiently large. However, the present minimal model was limited to include detailed mechanisms of hormone secretion. In the phenomenological model, hormone oscillations were intrinsically given without explicit consideration of the molecular details for generating the oscillations. Furthermore, we assumed that the amplitude and phase modulations (*f*_*σ*_ and *g*_*σ*_) were functions of glucose as a first approximation, although they can also depend on glucose change. Islets show an acute insulin secretion when glucose levels quickly change^34–36^. Thus, the minimal model may need to incorporate other relevant observations to serve as a more realistic simulator for glucose regulation.

The pulsatility of insulin secretion has functional advantages. Pulsatile insulin can suppress hepatic glucose production more effectively than constant insulin^37^, and it may prevent the desensitization of insulin receptors^38, 39^. Furthermore, it has been observed that the insulin pulsatility is diminished in diabetic patients^40^. This can be explained by the diminished pulse generation of β cells under diabetic conditions. However, loss of coordinated hormone secretions from multiple islets can also contribute to the diminished pulsatility of circulating insulin levels in blood. We demonstrated that the intra-islet network was special for controlling the inter-islet synchronization. Furthermore, the intra-islet network governed the phase coordination between glucagon, insulin, and somatostatin pulses (Fig. S9). The out-of-phase coordination between insulin and glucagon pulses should have physiological relevance. Hellman *et al*. have hypothesized that the inverse relation may enhance the insulin to glucagon ratio for hepatic glucose production^7^, and Menge *et al*. have reported that the inverse relation is disrupted in Type 2 diabetes^8^. Thus, our conclusion suggests that modifications of the local interactions between α, β, and δ cells under diabetic conditions can contribute to the abnormal global coordination of hormone secretions, in terms of (i) inter-islet synchronization, and (ii) phase relation between glucagon, insulin, and somatostatin pulses.

## Methods

The above differential equations were numerically integrated using the Euler method^41^ with a sufficiently small time step, Δ*t* = 0.0001.

## Electronic supplementarymaterial

Supplementary Information
