# Supplementary material for: A Local Counter-Regulatory Motif Modulates the Global Phase of Hormonal Oscillations
Source: Sci Rep. 2017 May 9;7:1602. doi: 10.1038/s41598-017-01806-0 (PMC5431656; doi:10.1038/s41598-017-01806-0)
Supplement: Supplementary file 1 — Supplementary Information [file 41598_2017_1806_MOESM1_ESM.pdf]

Supplementary Information

A Local Counter-Regulatory Motif Modulates the Global Phase of Hormonal Oscillations

Dong-Ho Park[1†], Taegeun Song[1†], Danh-Tai Hoang[1,2,3†], Jin Xu[1,4], and Junghyo Jo[1,4*]

[1]Asia Pacific Center for Theoretical Physics, Pohang, Gyeongbuk 37673, Korea

[2]Laboratory of Biological Modeling, National Institute of Diabetes and Digestive and Kidney Diseases, National Institutes of Health, Bethesda, Maryland 20892, United States of America

[3]Department of Natural Sciences, Quang Binh University, Dong Hoi, Quang Binh 510000, Vietnam

[4]Department of Physics, Pohang University of Science and Technology, Pohang, Gyeongbuk 37673, Korea

*Correspondence: jojunghyo@apctp.org

†These authors contributed equally to this work.

1. Islet model and glucose regulation

Here, we provide a complete description of hormone secretion and glucose regulation in Eqs. (3) and (4):

$$\dot{r}_{n\sigma} = \tau_r^{-1}\left[f_\sigma(G) - r_{n\sigma}^2\right] r_{n\sigma} + K\sum_{\sigma'} A_{\sigma\sigma'} r_{n\sigma'} \cos(\theta_{n\sigma'} - \theta_{n\sigma}), \qquad \text{(S1)}$$

$$\dot{\theta}_{n\sigma} = \omega_{n\sigma} - g_{n\sigma}(G)\cos\theta_{n\sigma} + K\sum_{\sigma'} A_{\sigma\sigma'} \frac{r_{n\sigma'}}{r_{n\sigma}} \sin(\theta_{n\sigma'} - \theta_{n\sigma}), \qquad \text{(S2)}$$

$$\dot{G} = \lambda\left[G_0 \sum_{n=1}^{N} r_{n\alpha}(1+\cos\theta_{n\alpha}) - G\sum_{n=1}^{N} r_{n\beta}(1+\cos\theta_{n\beta})\right] + I(t). \qquad \text{(S3)}$$

The islet model considers amplitude and phase modulations according to the glucose concentration. First, amplitude modulation controls the glucose-dependent hormone secretion of α, β, and δ cells (Fig. S1):

$$f_\alpha(G) = \frac{1}{2}\left[1 - \tanh\left(\frac{G - G_0}{5}\right)\right], \quad \text{(S3)}$$

$$f_\beta(G) = \frac{1}{2}\left[1 + \tanh\left(\frac{G - G_0}{5}\right)\right], \quad \text{(S4)}$$

$$f_\delta(G) = \frac{a_\delta}{2}\left[1 + \tanh\left(\frac{G - G_0 + \Delta G_0}{5}\right)\right], \quad \text{(S5)}$$

where $G_0 = 7$ mM, $\Delta G_0 = 2$ mM, and $a_\delta = 0.5$. The amplitude modulations are based on the observed glucose dose response of insulin, glucagon, and somatostatin secretions (*1*). Note that the U-shaped glucagon response under extremely high glucose (>20 mM) concentrations is not considered here. The somatostatin secretion of δ cells exhibits a lower glucose threshold than the insulin secretion. In addition, we parameterize the lower fraction of δ cells ($a_\delta < 1$) compared with two major populations of α and β cells.

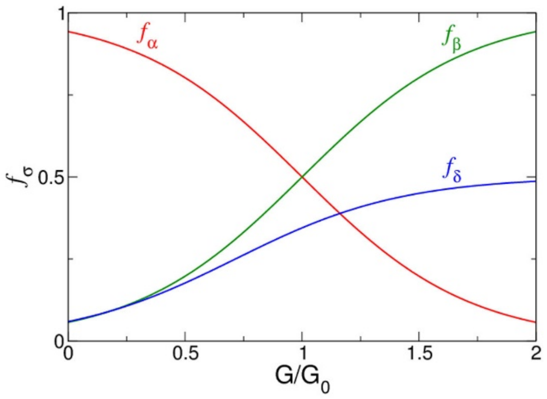

Fig. S1. Glucose-dependent amplitude modulation. α cells (f_α, red), β cells (f_β, green), and δ cells (f_δ, blue).

Second, phase modulation controls the duration of active/silent phases in the hormone pulses:

$$g_\alpha(G) = \mu(G - G_0), \quad \text{(S6)}$$

$$g_\beta(G) = \mu(G_0 - G), \quad \text{(S7)}$$

$$g_\delta(G) = \mu(G_0 - G) . \qquad \text{(S8)}$$

These phase modulations lead α cells to exhibit longer active phases at low glucose levels ($G < G_0$) and β and δ cells to exhibit longer active phases at high glucose levels ($G > G_0$). The glucose-dependent Ca^{2+} oscillations of islets showed that their active phases increase as glucose concentration increases (*2*). Since β cells are dominant (~80%) in islets, the result may support the phase modulation of β cells as shown in Fig. S2. However, direct experimental evidence still lacks for the phase modulations of α and δ cells due to the difficulty of cell identification. Here, we assumed that the phase modulation of α cells is opposite to β cells, while the phase modulation of δ cells is the same with β cells like their amplitude modulations. These descriptions complete the amplitude and phase dynamics in Eqs. (S1) and (S2).

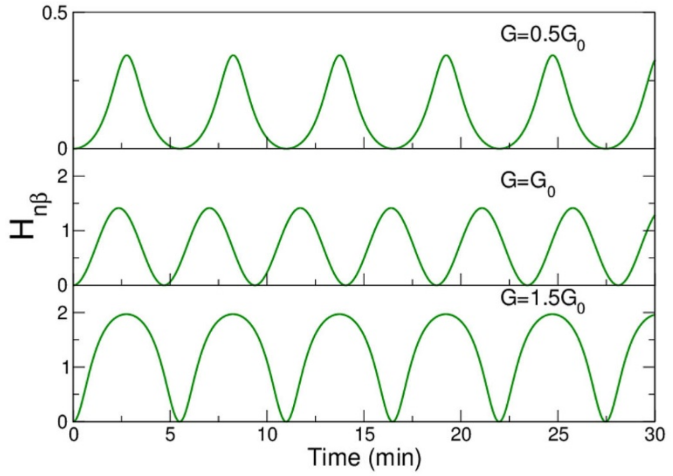

Fig. S2. Phase modulation. Glucose-dependent pulse shapes of insulin from a single β cell for various external glucose inputs.

One technical note is that because the negative amplitude $r_{n\sigma} = -\sqrt{f_\sigma}$ is another approximate solution of Eq. (S1), we avoid the negative amplitude through transformation as $r_{n\sigma} \to -r_{n\sigma}$ and $\theta_{n\sigma} \to \theta_{n\sigma} + \pi$ whenever a negative amplitude is confronted in simulations.

Each cell exhibits heterogeneous intrinsic phase velocities, $\omega_{n\sigma}$, of which periods follow a Gaussian distribution with a mean of 5 min and a standard deviation of 1 min. Table S1 summarizes our standard parameter values. The hormone and glucose dynamics was not

sensitive to initial conditions given a large number of total islets (*N*=200). In this study, we used $r_{n\sigma}(0) \in \{0.25, 0.75\}$, $\theta_{n\sigma}(0) \in \{0, 2\pi\}$, and $G(0) = 7$ mM.

Table S1. Standard parameter values.

Symbol	Values	Parameter
$2\pi / \omega_{n\sigma}$	5 ± 1 min	Intrinsic periods of islet cells
τ_r	1 min	Characteristic time for amplitude change
K	0.4 min^{-1}	Interaction rate between islet cells
μ	0.1 min^{-1}	Phase modulation rate
λ	1.0 min^{-1}	Hormone effectiveness rate
G_0	7 mM	Normal glucose concentration
ΔG_0	2 mM	Threshold shift of δ cells
a_δ	0.5	Amplitude reduction of δ cells
N	200	Total islet number

2. Parameter dependence of the islet model

Although we formulated the islet model constrained by experimental observations, exact information on parameter values is still lacking. Thus, we check the dependences of our conclusions.

(1) Interaction rate between islet cells, K

A too small K cannot distinguish the topological difference between islet-cell networks, while a too strong K induces strong nonlinear effects on the phase dynamics in Eq. (S2) and perturbs the principal spontaneous oscillations governed by the intrinsic frequency $\omega_{n\sigma}$. If K is sufficiently large ($K > 0.4$), the native network 121212 shows robust characteristics of small hormone consumption and small glucose fluctuations (Fig. S3).

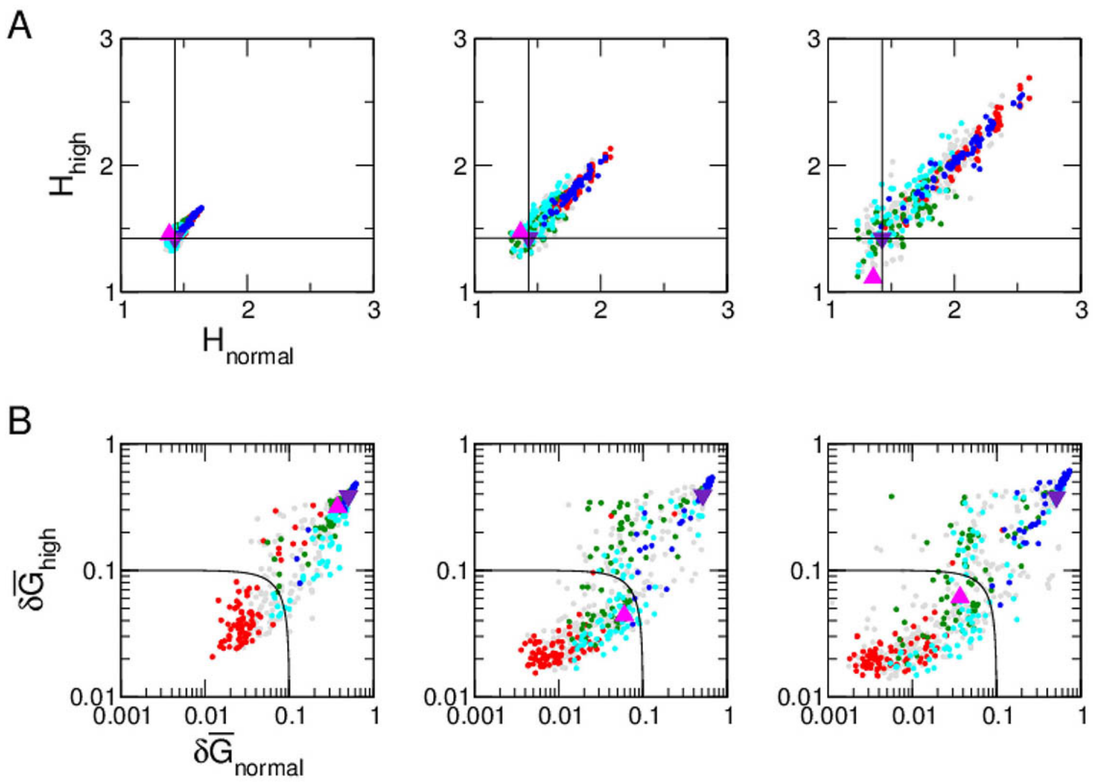

Fig. S3. Intra-islet coupling strength. Hormone consumption of 729 networks under normal ($I = 0$) and high ($I = 0.5G_0$) glucose conditions (A) and the temporal fluctuations of glucose (B) under various interaction rates, K = 0.1, 0.3, and 0.6, from left to right. The colors and notations are the same as in Fig. 2.

(2) Phase modulation rate, μ

Phase modulation through a pinning term in Eq. (S2) captures the glucose-dependent shapes of hormone pulses. Thus, the pinning term represents the response of islets to glucose perturbations. The oscillatory changes in glucose are then able to entrain islets to become synchronized. Here too weak pinning cannot induce inter-islet synchronization, while too strong pinning ($|\mu| > \omega_{n\sigma}$) will stop the oscillation of islet cells due to being stuck at $\theta_{n\sigma} = \cos^{-1}(\omega_{n\sigma} / \mu)$. At a lower pinning strength, network 000000 (no interaction between islet cells) starts to show inter-islet synchronization compared with network 121212 (Fig. S4). In addition, given the pinning strength ($0.07 < \mu < 0.2$), network 121212 generates different degrees of inter-islet synchronization for different external glucose inputs, I. The mean glucose concentration, $\overline{G}$, is independent of the pinning strength, but glucose fluctuations are highly correlated with inter-islet synchronization.

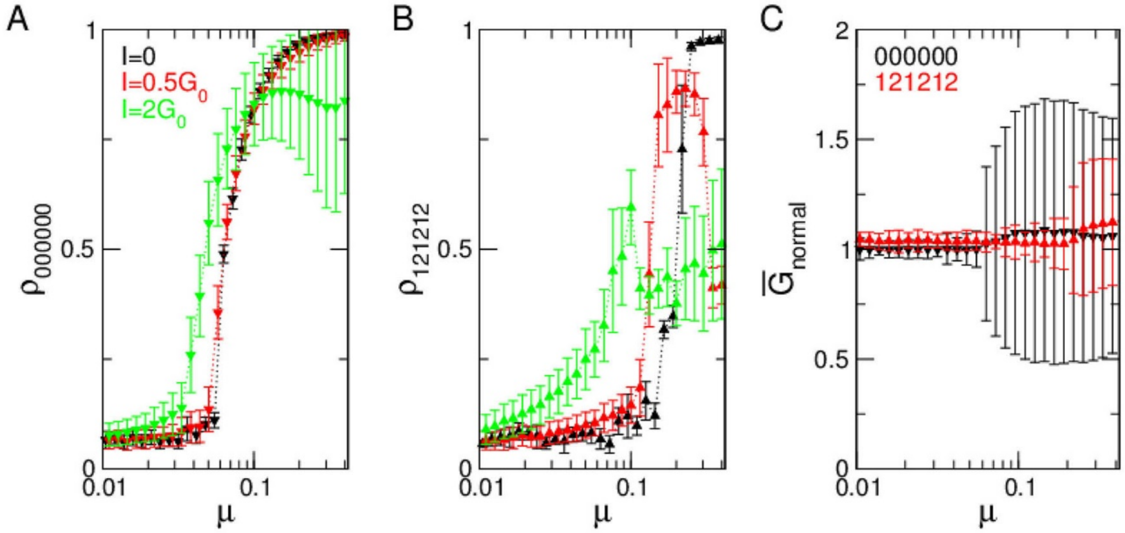

Fig. S4. Pinning strength. Inter-islet synchronization index, ρ, for networks (A) 000000 and (B) 121212 under external glucose inputs of $I = 0$ (black), $I = 0.5G_0$ (red), and $I = 2G_0$ (green). (C) Mean glucose concentration, $\overline{G}$, for networks 000000 (black) and 121212 (red) under normal glucose conditions ($I = 0$). Error bars represent the standard deviation.

(3) Amplitude reduction of δ cells, a_δ

Considering the minority of δ-cell populations, the value of a_δ is expected to be $0 < a_\delta < 1$. Glucose, hormone, and inter-islet synchronization profiles are not highly dependent on the variation of this parameter (Fig. S5). Two extreme networks, 120000 and 000202, correspond to ignorance ($a_\delta = 0$) and emphasis ($a_\delta > 1$) of the interactions of δ cells.

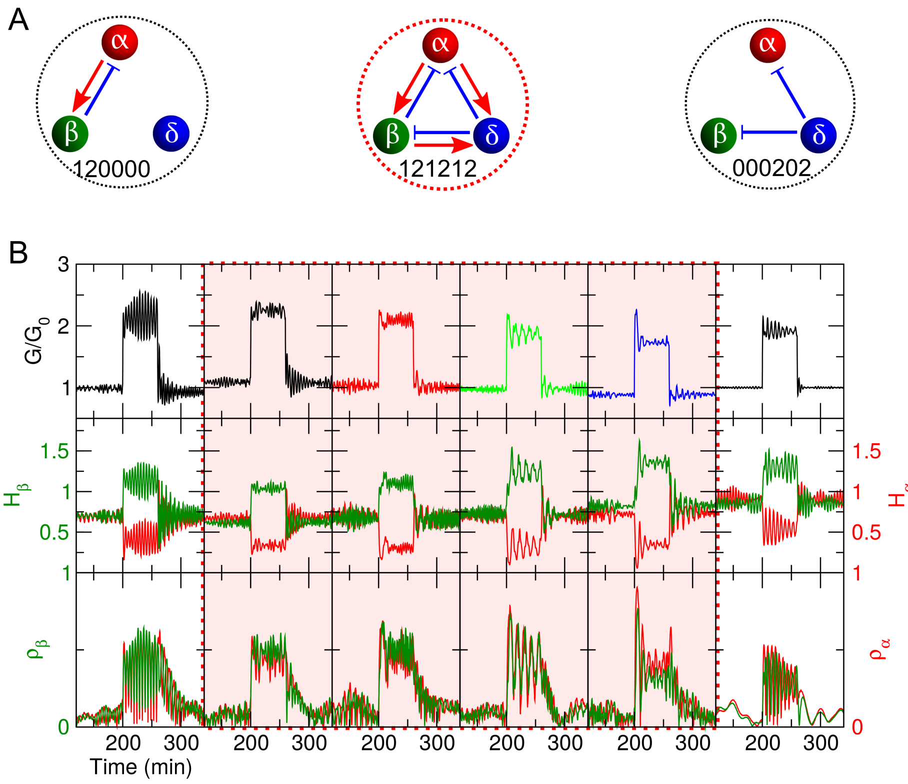

Fig. S5. Effect of δ-cell amplitude. (A) Two extreme networks, 120000 and 000202, ignoring and emphasizing the interactions of δ cells, respectively, compared with the native islet network, 121212. (B) Time traces of glucose, hormones, and inter-islet synchronization indices under a glucose stimulus of $I = 2G_0$ for 200 < Time < 260. The red-dotted box represents the results for network 121212 for a_δ= 0.1, 0.5, 1, and 2 from left to right. The leftmost column is the result of network 120000, and the rightmost column is the result of network 000202.

(4) Hormone effectiveness rate, λ

A too small λ fails to effectively regulate glucose in Eq. (2), while a too large λ diminishes the relative contribution of external glucose inputs, I, in Eq. (2) (Fig. S6).

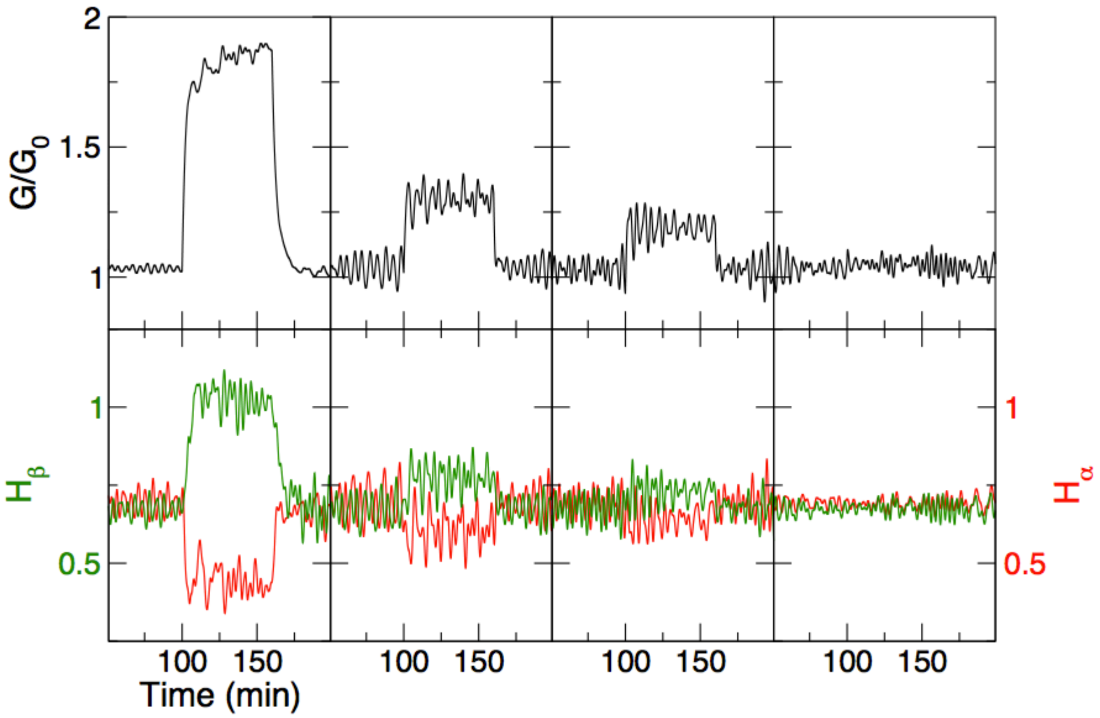

Fig. S6. Hormone effectiveness rate for glucose regulation. Time traces of glucose concentrations (upper) and hormones (lower) with various hormone effectiveness rates, λN= 0.2, 0.6, 1, and 10 min^{-1}, from left to right under a glucose stimulus of $I = 0.5G_0$ for 100 < Time < 160. For the simulation, network 121212 was used with total N=200 islets.

(5) Total islet number, N

As N increases, the fluctuation of glucose regulation decreases (Fig. S7). In addition, the initial condition dependence of the nonlinear dynamics is largely suppressed in the large N limit. For the analysis, we rule out an increase in the capacity of hormone secretions due to a larger N by normalizing the hormone effectiveness ($\lambda N = 1$).

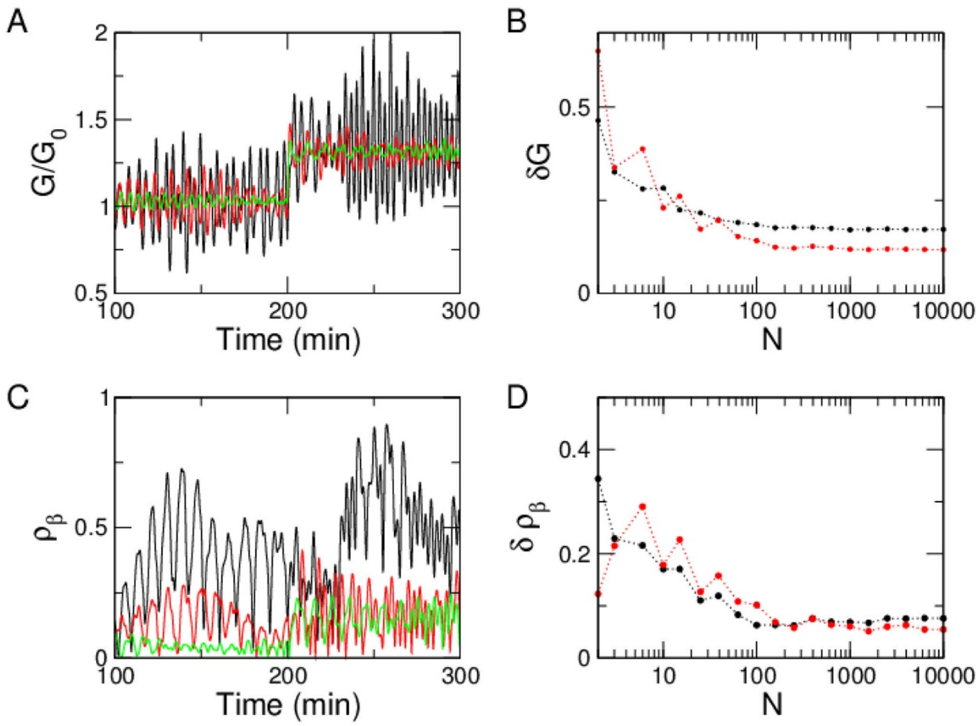

Fig. S7. Effects of islet number. (A) Temporal glucose profiles under a glucose stimulus ($I = 0.5G_0$) for Time > 200: N =10 (black), 100 (red), and 1,000 (green). (B) Temporal fluctuations of glucose the concentration for various numbers of islets before (black) and after (red) the glucose stimulus. Given the same protocol, (C) inter-islet synchronization index for β cells, and (D) its fluctuations. Dotted lines are drawn for guiding eyes. For the simulation, network 121212 was used.

(6) Time delay of hormone actions, τ

It takes time for the hormones secreted by the pancreas to act on the liver or peripheral tissues. The time delay can be simply considered by reformulating Eq. (2) as follows:

$$\dot{G} = \lambda N(G_0 H'_\alpha - G H'_\beta) + I(t), \qquad \text{(S9)}$$

$$\tau \dot{H}'_\sigma = H_\sigma - H'_\sigma, \qquad \text{(S10)}$$

where $\sigma \in \{\alpha, \beta\}$. The time delay, τ , may have a time scale of 1 minute, considering the circulation time of blood in the body. Such a short delay does not affect the regulation of glucose (Fig. S8). However, a long delay (τ =10 min) could not effectively regulate glucose with less hormone secretions.

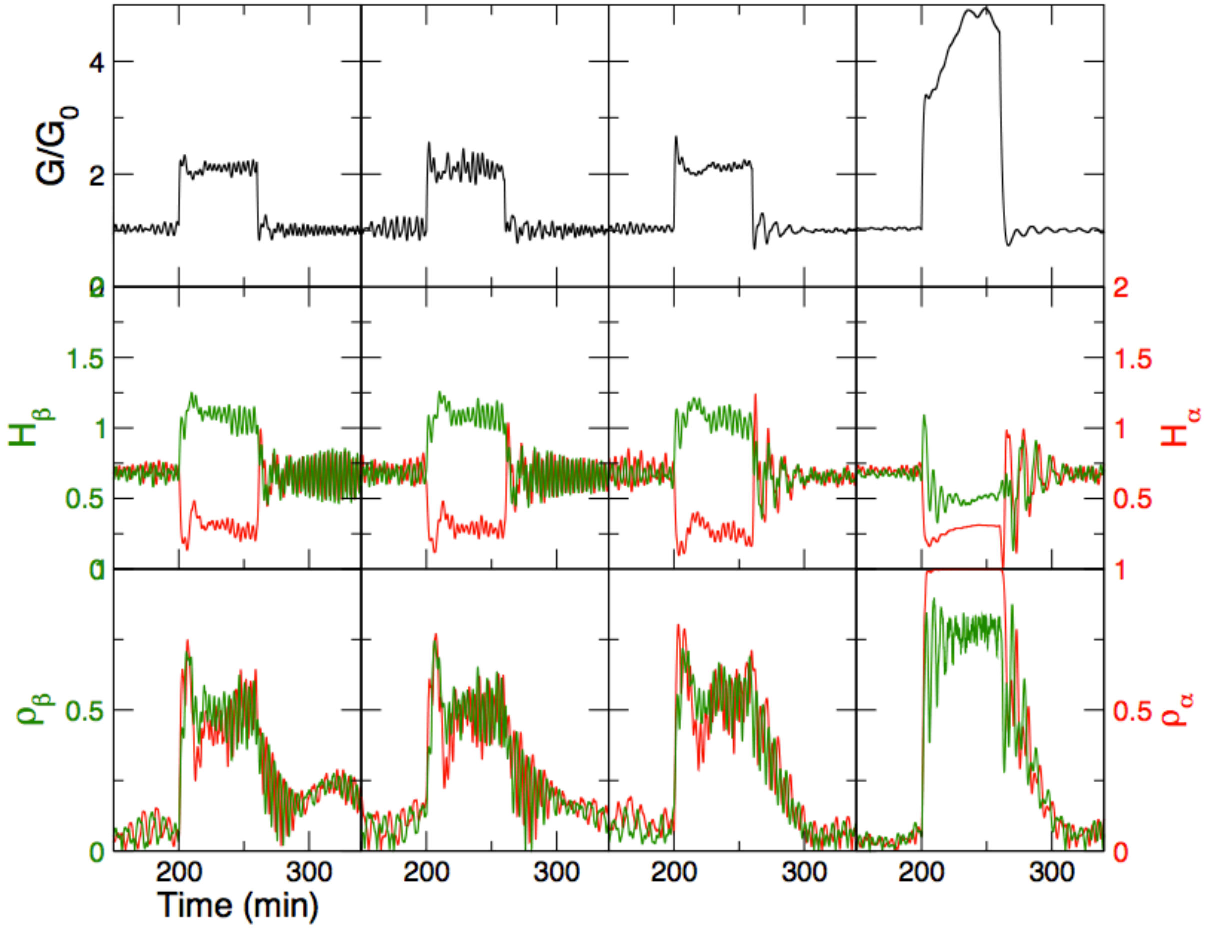

Fig. S8. Time traces of glucose, hormones, and the degree of synchronization under time delays. τ = 0, 0.1, 1 and 10 min from left to right. For the simulation, network 121212 was used with total N=200 islets.

3. Somatostatin oscillation and consumption

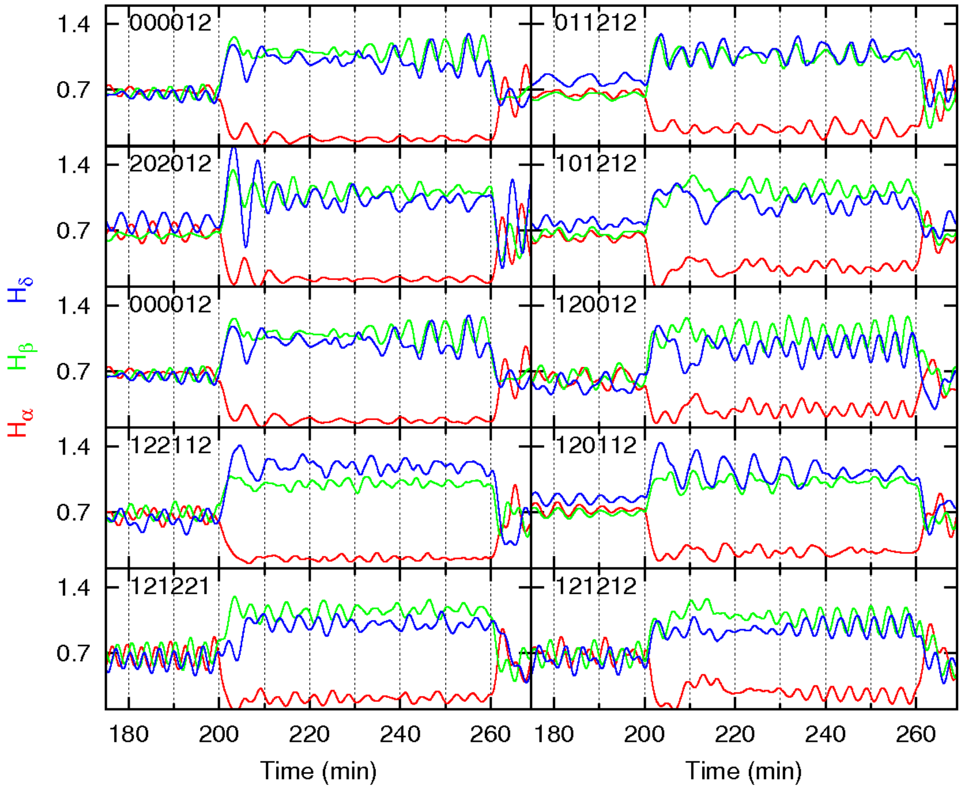

Fig. S9. Temporal hormone profiles for the 10 effective networks. Given the external glucose input $I = 2G_0$ for 200 < Time < 260, glucagon (H_α, red), insulin (H_β, green), and somatostatin (H_δ, blue) oscillated with time. Networks 121212 and 120012 showed clear phase coordination between the three hormones. For the simulation, standard parameter values were used (Table S1).

Table S2. Total hormone consumption and controllability of inter-islet synchronization.

Rank	Network	H_{normal}	$H_{\delta,\text{normal}}$	H_{high}	$H_{\delta,\text{high}}$	ΣH	$\rho_{\text{high}} - \rho_{\text{normal}}$
1	**120012**	1.249	0.575	1.388	0.942	4.154	0.307
2	101200	1.331	0.625	1.530	0.682	4.168	0.209
3	002102	1.312	0.626	1.574	0.711	4.223	0.209
4	202100	1.308	0.628	1.611	0.688	4.235	0.173
5	101202	1.423	0.621	1.528	0.677	4.249	0.206
6	**121212**	1.373	0.632	1.280	0.969	4.254	0.456
7	**000012**	1.347	0.651	1.293	0.996	4.287	0.228
8	210012	1.242	0.576	1.565	0.959	4.342	0.281
9	**121221**	1.363	0.612	1.376	0.994	4.345	0.323
10	002012	1.548	0.510	1.294	0.999	4.351	0.261
11	102102	1.512	0.611	1.533	0.717	4.373	0.238
12	**122112**	1.360	0.611	1.222	1.192	4.385	0.352
13	211202	1.529	0.602	1.468	0.798	4.397	0.290
14	102012	1.544	0.526	1.271	1.057	4.398	0.273
15	212100	1.258	0.594	1.558	1.011	4.421	0.316
16	120200	1.454	0.537	1.523	0.910	4.424	0.295
17	**202012**	1.333	0.774	1.298	1.026	4.431	0.214
18	122102	1.520	0.601	1.444	0.875	4.440	0.337
19	100000	1.616	0.548	1.435	0.867	4.466	0.215
20	002112	1.370	0.461	1.504	1.133	4.468	0.316
21	000002	1.561	0.559	1.488	0.864	4.472	0.252
22	**100012**	1.364	0.809	1.254	1.055	4.482	0.249
23	**101212**	1.284	0.772	1.421	1.013	4.490	0.276
24	120212	1.521	0.558	1.479	0.959	4.517	0.337
25	000112	1.307	0.626	1.559	1.052	4.544	0.285

Networks were sorted in ascending order of summed total hormone consumption at normal and high glucose conditions, $\Sigma H = H_{normal} + H_{\delta,normal} + H_{high} + H_{\delta,high}$, after selecting stable networks that showed small glucose fluctuations ($\overline{\delta G}^2_{\text{normal}} + \overline{\delta G}^2_{\text{high}} < 0.1^2$). The 10 effective networks in Table 1 have bold fonts.

4. Phase plane analysis for phase attractors

To understand the attractors of phase dynamics, we consider Eq. (S2) in a simplified setting in which the intrinsic angular velocities are identical ($\omega_{n\sigma} = \omega$), and the pinning term is off ($\mu = 0$). Note that the pinning term naturally becomes zero at normal glucose concentrations ($G = G_0$) in Eqs. (S6-8). Then, the phase equations for network 121212 are as follows:

$$\dot{\theta}_\alpha = \omega - K\frac{r_\beta}{r_\alpha}\sin(\theta_\beta - \theta_\alpha) - K\frac{r_\delta}{r_\alpha}\sin(\theta_\delta - \theta_\alpha), \qquad \text{(S12)}$$

$$\dot{\theta}_\beta = \omega + K\frac{r_\alpha}{r_\beta}\sin(\theta_\alpha - \theta_\beta) - K\frac{r_\delta}{r_\beta}\sin(\theta_\delta - \theta_\beta), \qquad \text{(S13)}$$

$$\dot{\theta}_\delta = \omega + K\frac{r_\alpha}{r_\delta}\sin(\theta_\alpha - \theta_\delta) + K\frac{r_\beta}{r_\delta}\sin(\theta_\beta - \theta_\delta). \qquad \text{(S14)}$$

Note that we removed the islet index, *n*, for simplicity. Because we are interested in the phase differences between α, β, and δ cells, relative phases ($x \equiv \theta_\alpha - \theta_\beta$ and $y \equiv \theta_\alpha - \theta_\delta$) can be defined. Using Eqs. (S12-14), we obtain the following:

$$\dot{x} = K\left[\frac{r_\beta}{r_\alpha} - \frac{r_\alpha}{r_\beta}\right]\sin x + K\frac{r_\beta}{r_\alpha}\sin y + K\frac{r_\delta}{r_\beta}\sin(x-y), \qquad \text{(S15)}$$

$$\dot{y} = K\frac{r_\beta}{r_\alpha}\sin x + \left[\frac{r_\delta}{r_\alpha} - \frac{r_\alpha}{r_\delta}\right]\sin y + K\frac{r_\beta}{r_\delta}\sin(x-y). \qquad \text{(S16)}$$

Depending on the amplitude, r_σ, different phase dynamics emerge, of approximately $r_\alpha > r_\beta, r_\delta$ at low glucose, $r_\beta > r_\alpha, r_\delta$ at high glucose, and $r_\alpha \approx r_\beta \approx r_\delta$ at normal glucose. Under the given glucose conditions, the phase dynamics exhibit single and triple attractors under low/high and normal glucose (Fig. S10).

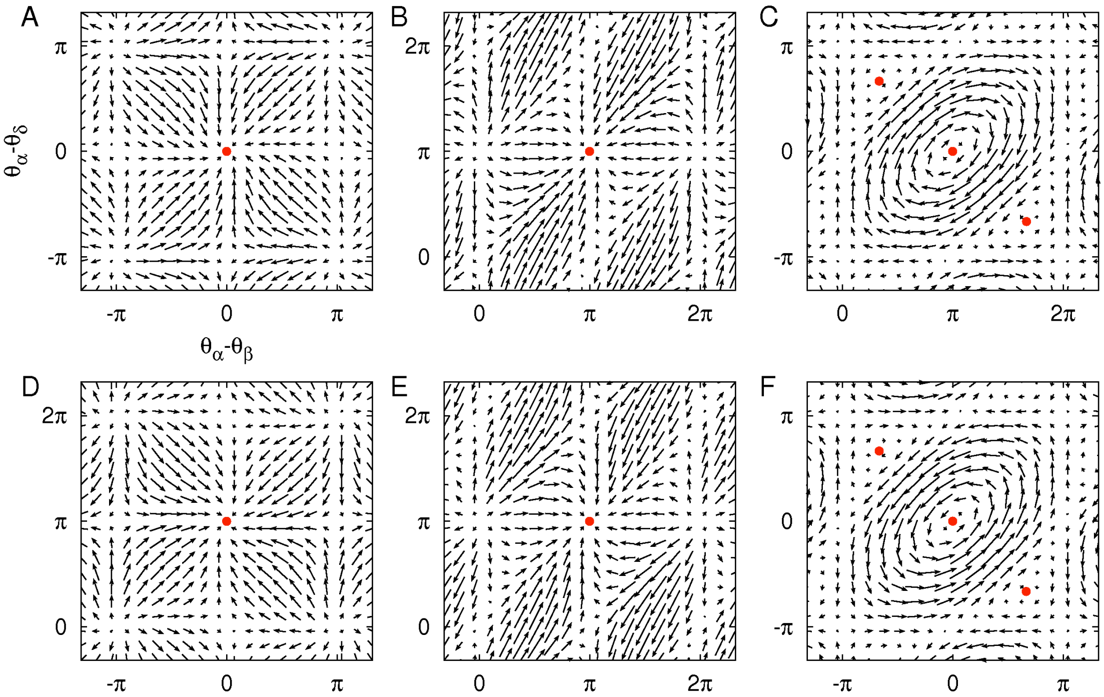

Fig. S10. Vector flows in phase dynamics. (A, D) $r_\alpha = 1, r_\beta = r_\delta = 0.2$, (B, E) $r_\beta = 1, r_\alpha = r_\delta = 0.2$, (C, F) $r_\alpha = r_\beta = r_\delta = 1$ for network 121212 (A, B, C) and network 122112 (D, E, F).

5. Low glucose challenge

Pancreatic islets regulate not only high glucose but also low glucose. Therefore, we challenged islets by lowering glucose concentrations with a negative glucose influx ($I < 0$). Controllable inter-islet synchronization emerges at low glucose levels (Fig. S11).

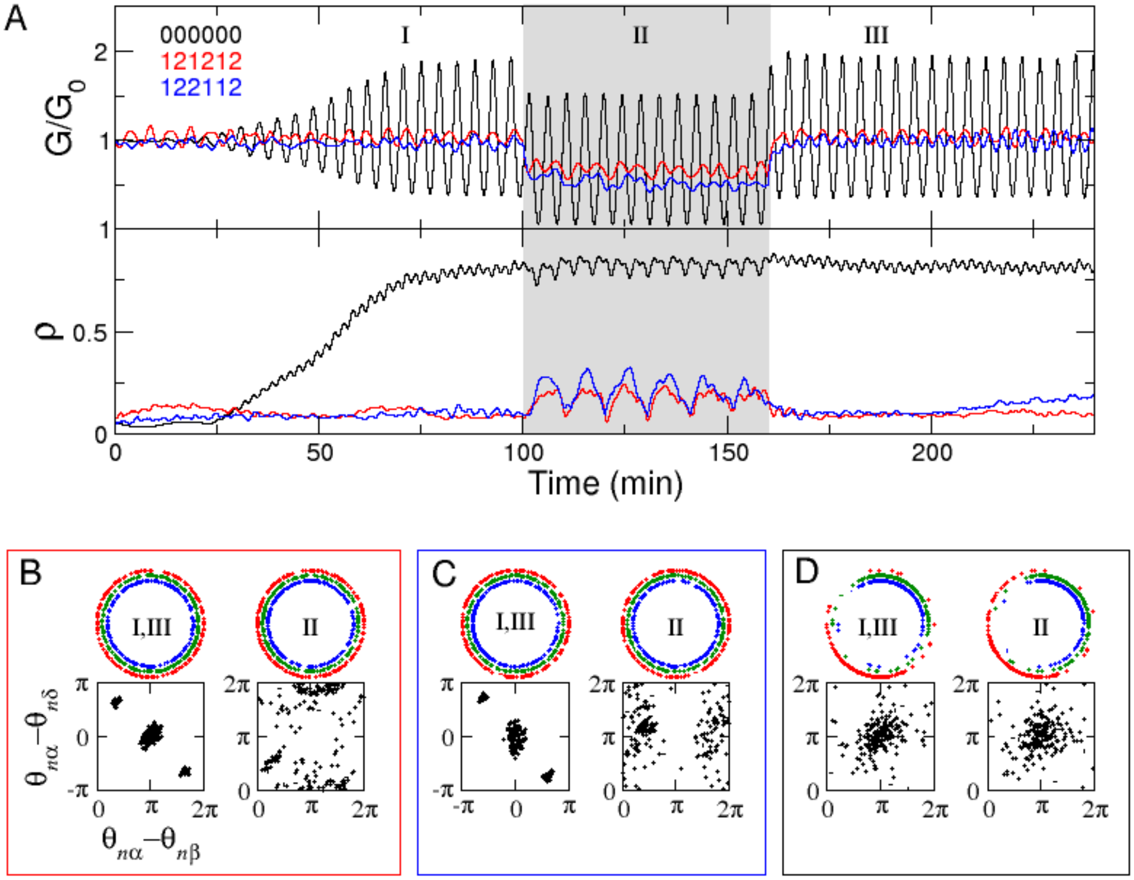

Fig. S11. Controllable inter-islet synchronization and phase coordination between islet cells. (A) Glucose regulation and inter-islet synchronization for networks 121212 (red), 122112 (blue) and 000000 (black), given the external glucose input ($I = -0.5G_0$) during 100 < t < 160. Phase snapshots of α, β, and δ cells under different glucose conditions (regimes I, II, and III) for (B) networks 121212, (C) 122112, and (D) 000000. Upper panel: absolute phases ($\theta_{n\alpha}, \theta_{n\beta}, \theta_{n\delta}$) of α (red), β (green), and δ (blue) cells in the pancreas consisting of 200 islets. Lower panel: phase differences ($\theta_{n\alpha} - \theta_{n\beta}, \theta_{n\alpha} - \theta_{n\delta}$). The axis range was adjusted to show distinct attractors considering 2π periodicity.

6. Population model

Real islets are composed of populations of islet cells rather than single α, β, and δ cells within each islet. Therefore, we consider populations of islet cells with a known composition and organization (*3*). In the population model, each cell has different nearest neighbors. The interaction follows exactly the same pattern as the single-cell model, but one has to consider autocrine interactions in which the same cell types interact with each other in this case. We used positive autocrine interactions, as in a previous study (*4*). We confirmed that controllable synchronization is also realized in the population model (Fig. S12).

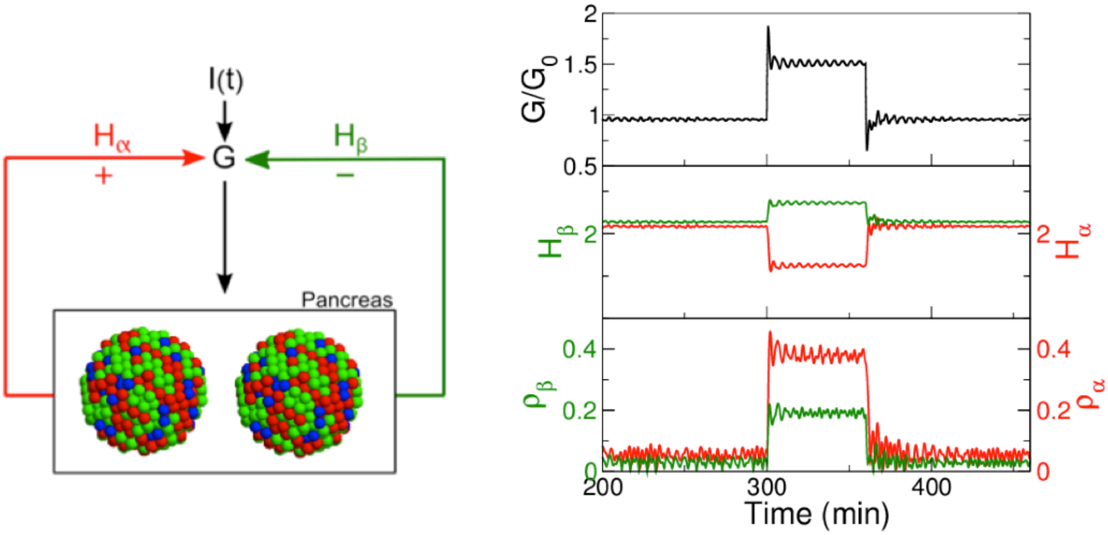

Fig. S12. Population model and controllable synchronization. The pancreas is composed of 1,000 islets, and each islet has 1,357 cells (30% α, 60% β, and 10% δ cells) that are organized for a partial mixing structure. Given glucose stimulus ($I = 3G_0$) for 300 < Time < 360, total 1,000×1,357 islet cells regulate glucose level. Plotted are glucose change, hormone secretions, and degrees of synchronization between α cells in different islets and between β cells in different islets.

7. Noisy glucose stimulus

To probe noise dependence for glucose regulation, we introduced noise in glucose input:

$$I(t) = I_1 + I_2\,\xi(t), \qquad \text{(S17)}$$

where $\xi(t)$ is a white noise with $\langle \xi(t) \rangle = 0$ and $\langle \xi(t)\xi(t') \rangle = \delta(t - t')$. After conducting the stochastic simulation, we confirmed that the noise made glucose changes and synchronization indices more jiggling (Fig. S13), but it did not change our main conclusion about the effectiveness of network 121212 for glucose regulation (Fig. S14). However, we did not see exceptional noise tolerance of network 121212 compared with other network motifs.

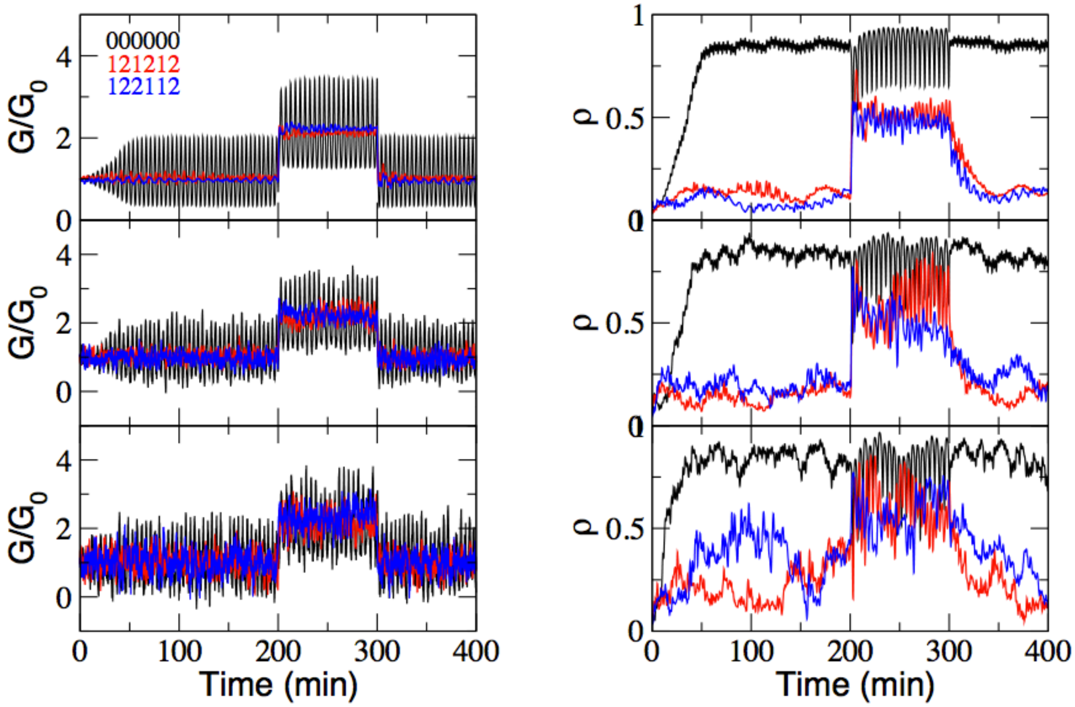

Fig. S13. Noisy glucose stimulus. Glucose (left) and inter-islet synchronization index (right) changes under noisy glucose stimuli: $I_2 = 0$ (top), $0.2G_0$ (middle), and $0.4G_0$ (bottom). To have normal and high glucose conditions, $I_1 = 2G_0$ was infused for 200 < Time < 300, otherwise $I_1 = 0$. Networks 000000 (black), 121212 (red), and 122112 (blue). The standard parameter values were used for this simulation.

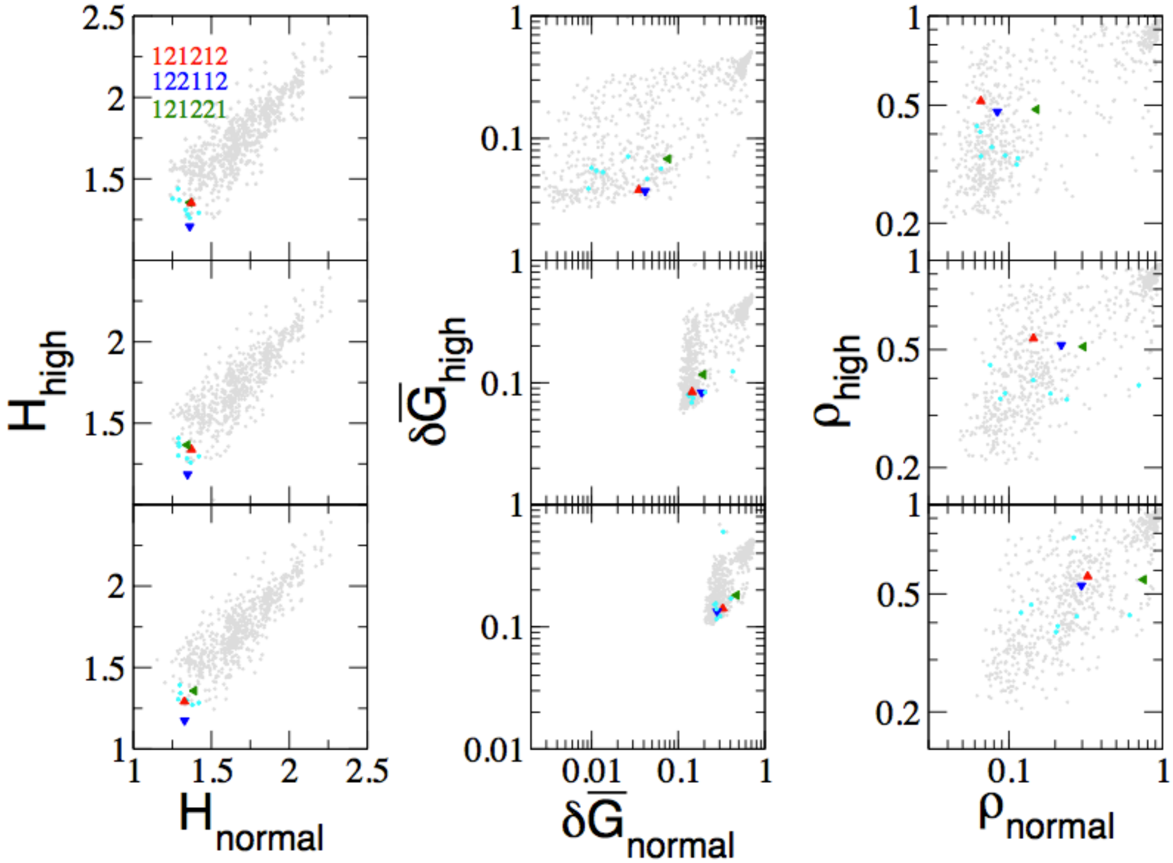

Fig. S14. Effective networks under noisy conditions. Hormone consumptions, glucose fluctuations, and inter-islet synchronizations under normal ($I_1 = 0$) and high ($I_1 = 2G_0$) glucose conditions with noisy glucose stimuli: $I_2 = 0$ (top), $0.2G_0$ (middle), and $0.4G_0$ (bottom). Networks 121212 (red), 122112 (blue), 121221 (green), other seven effective networks (cyan), and remaining 719 networks (gray). The standard parameter values were used for this simulation.

8. Oscillatory glucose stimulus

To probe the islet response under oscillatory glucose stimulus, we introduced an oscillating glucose input:

$$I(t) = I_1 \cos(\omega_{ext} t), \quad \text{(S17)}$$

where the external driving frequency was set as $2\pi / \omega_{ext} = 5$ min. If the driving frequency is too fast or too slow compared with the intrinsic frequencies $\omega_{n\sigma}$ of islet cells, the oscillatory stimuli cannot entrain islets. When the driving amplitude, I_1, was sufficiently large, the hormone secretions from islets were entrained to the external driving (Fig. S15). Here, networks 121212 and 122112 could resist to be entrained by small oscillatory glucose stimuli unlike network 000000.

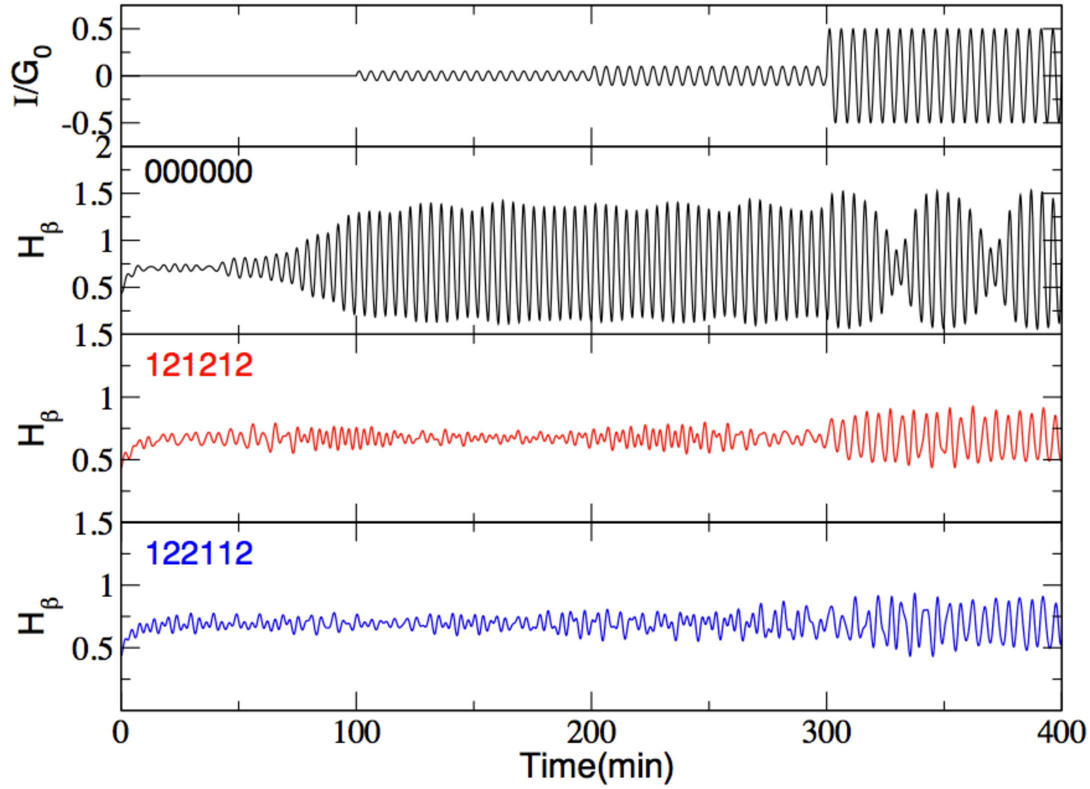

Fig. S15. Oscillatory glucose stimulus and hormone secretion. Hormone secretion of networks 000000 (black), 121212 (red), and 122112 (blue) under oscillatory glucose stimuli: $I_1 = 0 (0 < \text{Time} < 100)$, $0.05G_0$ $(100 < \text{Time} < 200)$, $0.1G_0$ $(200 < \text{Time} < 300)$, and $0.5G_0$ $(300 < \text{Time} < 400)$. The standard parameter values were used for this simulation.

References

1. E. Gylfe, Glucose control of glucagon secretion—'There's a brand-new gimmick every year.' *Upsala Journal of Medical Sciences* **121**, 120-132 (2016).
2. J. Fernandez, M. Valdeolmillos, Synchronous glucose-dependent $[Ca^{2+}]_i$ oscillations in mouse pancreatic islets of Langerhans recorded in vivo. *FEBS Letters* **477**, 33-36 (2000).
3. D.-T. Hoang *et al*., A conserved rule for pancreatic islet organization. *PLoS ONE* **9**, e110384 (2014).
4. D.-T. Hoang, M. Hara, J. Jo, Design principles of pancreatic islets: glucose-dependent coordination of hormone pulses. *PLoS ONE* **11**, e0152446 (2016).
